# Definitions, Foundations and Associations of Physical Literacy: A Systematic Review

**DOI:** 10.1007/s40279-016-0560-7

**Published:** 2016-06-30

**Authors:** Lowri C. Edwards, Anna S. Bryant, Richard J. Keegan, Kevin Morgan, Anwen M. Jones

**Affiliations:** 1 Cardiff School of Sport, Cardiff Metropolitan University, Cyncoed Campus, Cardiff, CF23 6XD UK; 2 Faculty of Health, Research Institute for Sport and Exercise, University of Canberra, Bruce, ACT Australia

## Abstract

**Background:**

The concept of physical literacy has stimulated increased research attention in recent years—being deployed in physical education, sport participation, and the promotion of physical activity. Independent research groups currently operationalize the construct differently.

**Objective:**

The purpose of this systematic review was to conduct a systematic review of the physical literacy construct, as reflected in contemporary research literature.

**Methods:**

Five databases were searched using the preferred reporting items for systematic reviews and meta-analyses (PRISMA) guidelines for systematic reviews. Inclusion criteria were English language, peer reviewed, published by March 2016, and seeking to conceptualize physical literacy. Articles that met these criteria were analyzed in relation to three core areas: properties/attributes, philosophical foundations and theoretical associations with other constructs. A total of 50 published articles met the inclusion criteria and were analyzed qualitatively using inductive thematic analysis.

**Results:**

The thematic analysis addressed the three core areas. Under definitions, core attributes that define physical literacy were identified, as well as areas of conflict between different approaches currently being adopted. One relatively clear philosophical approach was prominent in approximately half of the papers, based on a monist/holistic ontology and phenomenological epistemology. Finally, the analysis identified a number of theoretical associations, including health, physical activity and academic performance.

**Conclusions:**

Current literature contains different representations of the physical literacy construct. The costs and benefits of adopting an exclusive approach versus pluralism are considered. Recommendations for both researchers and practitioners focus on identifying and clearly articulating the definitions, philosophical assumptions and expected outcomes prior to evaluating the effectiveness of this emerging concept.

**Electronic supplementary material:**

The online version of this article (doi:10.1007/s40279-016-0560-7) contains supplementary material, which is available to authorized users.

## Key Points

**Table:** 

This paper is the first to provide a systematic review of core attributes of the physical literacy construct, including the defining properties of physical literacy, the philosophical foundations and the theoretical associations of the construct.
An implication for theory development and research is the need for transparency and tolerance with different approaches to physical literacy.
Implications for applied practice include ensuring clarity of theoretical descriptions and phrases so that these can be translated clearly into a practical setting.

## Introduction

The concept of physical literacy has gained prominence in recent years, in many different countries [1–5], with scientific papers on the subject increasing from one in 1998 to 29 in 2014 [6]. Educational organizations and researchers around the world have argued that physical literacy should be given the same educational value as literacy and numeracy [1, 7, 8]. While there are many organizations, research groups and governments currently promoting physical literacy interventions around the world, the definitions adopted differ [4]. This chaotic situation may undermine the meaningful measurement of physical literacy, interpretation of findings, and prevents any meaningful accrual/agglomeration of research findings [6].

Many definitions of physical literacy refer to lifelong participation in physical activity [9–11]. According to the World Health Organization (WHO), physical activity is defined as “any bodily movement produced by skeletal muscles that requires energy expenditure” [12]. The importance of distinguishing between physical literacy and physical activity is emphasized by Whitehead [13], who offered the definition of physical literacy as “the motivation, confidence, physical competence, knowledge and understanding to value and take responsibility for engagement in physical activities for life” [13]. Physical literacy has become a key focus of physical activity [14] and, as such, physical literacy is arguably an antecedent of physical activity, while also being developed through physical activity. Physical activity has been consistently demonstrated to generate considerable health benefits, such as reducing the likelihood of cardiovascular disease, diabetes and cancer [15]. Hence, the promotion of physical literacy has been identified as a key opportunity to generate significant health benefits [16] in both children [17] and adults [18]. Furthermore, improving individuals’ physical activity (and by association, physical literacy) may have the potential to reduce financial expenses to healthcare systems [19–21] and increase academic performance [22–24]. This situation makes it particularly important to clarify the meaning of physical literacy, ways of developing it and the likely consequences of promoting it.

### A Debated Definition

Physical literacy has been referred to, in a metaphorical sense, as developing literacy within a physical setting, synonymous to reading and writing, and specific to the culture in which individuals live [1]. Other definitions of physical literacy focus solely on developing physical competencies such as fundamental movement skills (FMS), motor development, running speed and ‘exergames’ [18, 25–35]. Additionally, there exists a range of overlapping terms, such as movement literacy [36], aesthetic literacy [37], health literacy [10] and games literacy [38]. Not only are there different ways of defining and operationalizing physical literacy, there are also a range of competing constructs that may occupy very similar conceptual space. In this respect, the concept of physical literacy may be in danger of becoming diluted, redundant or meaningless.

In order for a coherent research tradition to develop, it is necessary to reach a level of clarity and transparency in relation to core constructs and, indeed, a level of consensus between researchers [39]. When a study claims to have measured or promoted physical literacy, and supported or refuted the associated theoretical claims, it is important to know exactly what was measured and what claims were tested. A further reason that research paradigms can degenerate is when there is no clarity regarding the underlying philosophy, or assumptions regarding the nature of the phenomena being studied [39]. Whitehead has proposed relatively detailed philosophical groundings for physical literacy, drawing from phenomenology, existentialism and monism [40]. It is possible that some researchers consider these philosophies as idealistic and complex [41]. However, if researchers are unable to articulate the hypothetical mechanisms explaining how concepts influence one another, then it is possible that no scientific theory is being tested by research, only relatively arbitrary/baseless and unscientific predictions. Hence, as well as understanding the defining properties of physical literacy and the underpinning philosophy, the final step in articulating a coherent ‘paradigm’ is to detail the theoretical associations and predictions offered by the theory. Such predictions could then be operationalized and tested, and these tests would be instructive as to whether the underpinning theory, assumptions and definitions are valid.

It is evident from the above discussion that there are a number of inconsistencies surrounding physical literacy; however, these contrasting arguments have not yet been evaluated systematically. To remedy this situation, the current paper adopted the systematic review methodology with a view to summarizing, appraising and communicating relevant research [42]. Systematic reviews utilize explicit, rigorous and transparent methods in order to minimize bias and offer a complete, coherent overview of contemporary knowledge on a topic [43].

### Aims and Research Questions

The purpose of this systematic review was to collate, analyze and evaluate the core attributes of the physical literacy construct, as reflected in contemporary research literature (up to March 2016). This paper will explore and critically discuss the following three research questions: What are the (a) defining attributes; (b) philosophical underpinnings; and (c) theoretical associations of physical literacy in peer reviewed, published papers that attempt to define the concept?

## Methods

### Search Strategy

An electronic search strategy was employed using the following databases: (i) SPORTDiscus; (ii) MEDLINE (via PubMed); (iii) Scopus; (iv) ScienceDirect; and (v) Education Research Complete. No particular start date was adopted and the last search was conducted on 22 March 2016. These education, sport and health databases are suitable for the topic and increase the probability that all relevant studies have been located [44, 45]. The Boolean logic combinations search strategy was adopted within the electronic databases, including ‘physical literacy’, with physical education (PE), health literacy, movement literacy, fundamental movement skills, games literacy, gross motor skills, and kinesthetic literacy. Inverted commas were inserted around the term ‘physical literacy’ to ensure searches would find papers in relation to physical literacy as opposed to searches related to ‘physical’ and ‘literacy’. ‘English’ and ‘peer-reviewed’ filter boxes were marked on all searches to ensure only these papers would appear in the results (see electronic supplementary material Appendix S1).

### Eligibility Criteria

The criteria for inclusion in this systematic review were (i) papers with a peer reviewed, published status; and (ii) publications in the English language until the date last searched, i.e. 22 March 2016. To address the aims and research questions of the study, the following exclusion criteria were adopted: (i) papers not covering the definition, philosophy or associations of physical literacy; (ii) papers that used physical literacy in the title, keywords or abstract, but made no reference to physical literacy in the full body of text; (iii) book chapters, book reviews and book synopses; (iv) conference reports and readings; and (v) editorials and forewords. As a result of adopting the inclusion and exclusion criteria, only papers from 2000 onwards met the eligibility criteria.

**Fig. 1:**
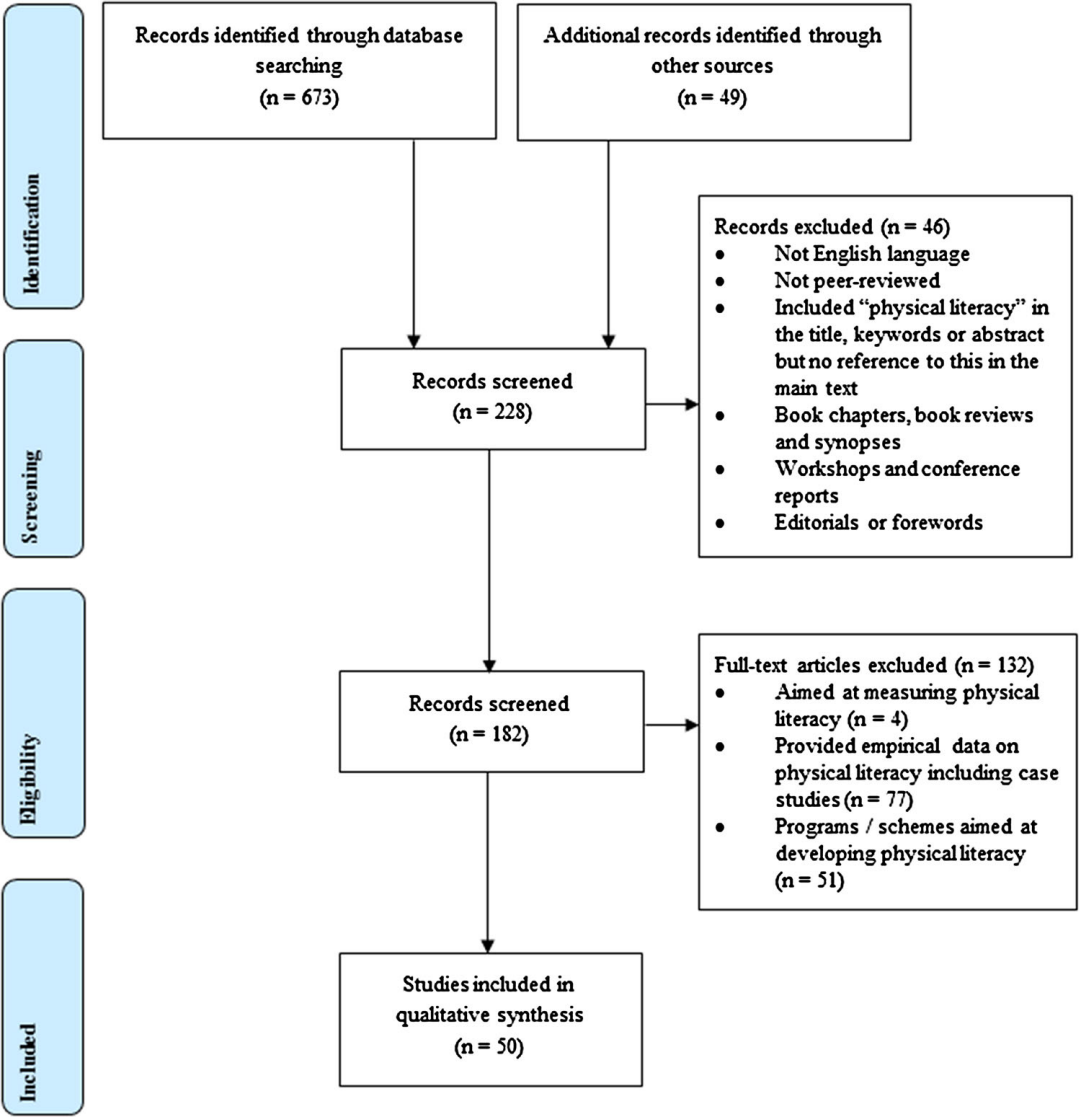
PRISMA flow diagram showing the process of study identification and selection [46]. *PRISMA* preferred reporting items for systematic reviews and meta-analyses

The authors followed the preferred reporting items for systematic reviews and meta-analyses (PRISMA) guidelines, an evidence-based checklist (see electronic supplementary material Appendix S2) for authors to use when reporting systematic reviews and meta-analyses [46]. In accordance with the PRISMA procedures, all duplicate papers, i.e. the same paper from different search engines, were removed. After 145 duplicates were removed, non-duplicated papers were read thoroughly and were considered either suitable or unsuitable following the inclusion and exclusion criteria (records were kept of this process). To minimize the risk of bias in individual studies, the authors followed the following two steps. First, data were extracted and analyzed in an inductive manner only if they pertained to the definition, core philosophy, or conceptual association of physical literacy. Second, to reduce reviewer selection bias and thus increase the reliability of selection, two reviewers independently examined and selected the applicable papers for the review, and a mutual agreement was made between reviewers as to whether or not they met basic inclusion criteria [47]. This process was documented and any discrepancies between reviewers were resolved by consensus and/or discussion with the third investigator. Records were kept of this process, with a 92 % agreement prior to discussion and 100 % post discussion. During the data analyzing process, the research team performed the following roles: the main analyst (LE), one coanalyst (AB), two consensus validators (KM and AJ) and one external critical colleague (RK). After this thorough process, and consistent with the exclusion criteria, a total of 50 papers were included in the review (see Fig. 1), including 36 different unique first authors.

### Data Extraction and Synthesis

Thematic coding was used to identify distinct categories from the papers in the analysis. Thematic coding is a method of identifying common themes within passages of texts with a purpose of data retrieval [48]. The coding process goes beyond only considering key words or phrases directly from the text, but concentrates on describing both implicit and explicit concepts within the data [49]. For the purpose of this study, a two-step process was performed: first using basic coding techniques to identify the general themes (see electronic supplementary material Appendix S3), followed by thorough and interpretative coding, which highlighted more specific trends in the data [50]. This coding procedure allowed replication and transparency of data synthesis [51].

Qualitative synthesis using thematic analysis was conducted on the 50 applicable papers as the systematic review was concerned with meanings and semantics and not quantitative data. Thematic analysis is used frequently as a form of analysis in qualitative research and includes analytical examination and recording themes within data [52]. It was essential that the analyst was familiar with the content of the data through repeating reading of each text [53]. Next, initial codes were generated inductively under the headings of (a) properties of physical literacy; (b) philosophical underpinning; and (c) proposed associations. Once organized into three general themes, or ‘higher-order themes’ (see Table 1), an in-depth coding process occurred to identify the core categories and subthemes related to the higher-order theme [52]. The in-depth process included reading through every paper several times and highlighting key words and phrases in relation to the three higher-order themes, as outlined above. Finally, core categories and subthemes were reviewed and clearly defined before producing the final framework. The analysis summarized in Table 1 demonstrates the hierarchical structure that was deployed, building from raw codes to core categories, subthemes and higher-order themes. The tabulated information permits readers and authors to clearly identify the progression to a higher-order theme as well as the frequency of appearance of each core category.

**Table 1:** Physical literacy hierarchical structure, including core categories, subthemes and higher-order themes

Core categories^a^	Subthemes	Higher-order themes
Confidence (26)	Affective	Properties of physical literacy
Motivation (23)		
Self-esteem (4)		
Knowledge and understanding of activities (16)	Cognitive	
Knowledge and understanding of healthy and active lifestyles (13)		
Value and take responsibility for physical activity (2)		
Movement capacities (22)	Physical capabilities	
Motor skill competence (18)		
Physical competence (12)		
Fundamental movement skills (8)		
Purposeful physical pursuits (6)		
Throughout the lifespan (19)	Progression/developmental pathway	
Unique journey (7)		
Long-Term Athlete Development model (5)		
Children (13)	Target audience	
All can develop physical literacy (3)		
Importance for adults (3)		
Read/interact with environment (14)	Holistic concept	
Movement with poise and economy (5)		
Health literacy (3)	Related constructs	
Aesthetic literacy (1)		
Develop whole person (15)	Ontological assumptions	Philosophical underpinning
Embodied (16)		
Monism (16)		
Human disposition (8)		
Phenomenology (8)	Epistemological assumptions	
Existentialism (7)		
Meaningful experience (5)	Pedagogical implications	
Pragmatic reality (3)		
Not a pedagogical model (2)		
Physical activity (22)	Behavioral characteristics	Associations and relationships
Health behaviors (13)		
Engage, enthuse and enjoy (13)	Psychological, social and attitudinal	
Support from significant others (10)		
Cognitive/academic performance (4)		
Physical education (38)	Contextual	
Sport sector (8)		

^a^Numbers in parentheses represent the number of papers that referred to the core categories apparent, of a possible 50 papers

## Results

### Summary of Studies

The number of papers that were identified, screened, and considered for eligibility are summarized in Fig. 1 [46]. Table 1 provides an overview of the core categories, subthemes and higher-order themes used for the analysis on the 50 papers for this systematic review.

### Qualitative Synthesis

The analysis identified a total of 694 codes, which were organized into 37 core categories and 13 subthemes; these were then organized into higher-order themes representing the three aspects of our research question. The following section will review these three higher-order themes from the analysis; namely, properties of physical literacy, the philosophical underpinnings, and the theoretical associations of physical literacy from the perspectives of the papers being discussed. Each subtheme is defined and explained, and the core categories that constitute the subthemes are discussed.

#### Properties of Physical Literacy

For this higher-order theme, 22 core categories were evidenced under the following seven subthemes: affective, cognitive, physical capabilities, progression/developmental pathway, target audience, holistic concept and related constructs.

##### Affective

The three core categories that were adopted under the ‘affective’ subtheme included confidence, motivation and self-esteem. The affective domain describes one’s motivation and confidence in relation to physical activities [16]. Individuals who are lacking in confidence, motivation and self-esteem are more likely to have lower incentive to participate in physical activity [16]. As identified in Table 1, the analysis reveals that the affective element was adopted in the papers more frequently than the cognitive and physical elements; confidence was adopted in 26 papers and motivation was adopted in 23 papers, compared with knowledge and understanding of activities (16 papers), knowledge and understanding of healthy and active lifestyles (13 papers) and physical competence (evident in only 12 papers). Four papers suggested that developing along one’s physical literacy journey can impact on attitudinal factors, including encouraging self-esteem in a physical environment [54–57]. Consistent with the philosophical underpinnings of physical literacy, Whitehead proposes that an awareness of embodiment and interacting with the physical environment stimulates positive self-esteem and self-confidence [13, 54, 58]. The reasoning is that individuals with high self-esteem are prone to engage fully with physical activities, whereas individuals with low self-esteem are likely to avoid unnecessary physical activities as a method of evading potential embarrassment or disappointment [13].

##### Cognitive

The cognitive properties of physical literacy centered on knowledge and understanding, and were evident in 29 papers. In general terms, the basis of what constitutes a literate individual in any domain is an acquired knowledge and understanding in a variety of settings [3, 59, 60]. Three core categories were deployed in the cognitive subtheme; specifically, knowledge and understanding of activities, knowledge and understanding of healthy and active lifestyles and the ‘value to take responsibility for physical activity’. A thorough knowledge and understanding of activities is characteristic of a literate sports person, particularly concerning the sports’ rules, traditions, and values [61], while knowledge and understanding of health and active lifestyle is a means to cognitively create a sound underpinning and awareness to value participating in a physically active lifestyle [9, 40, 54]. Furthermore, a total of 38 of the 50 papers in the analysis referred to Whitehead’s definition of physical literacy, which is inclusive of the phrase “value and take responsibility for maintaining purposeful physical pursuits/activities throughout the life-course” [13], which was perceived vital to the cognitive domain.

##### Physical Capabilities

Physical capabilities refers to the physical domain and was divided into five core categories; namely, movement capacities, motor skill competence, physical competence, FMS and purposeful physical pursuits.

Physical competence was coded in 12 papers and was defined as one’s ability to move with competence in a wide variety of activities [3, 13, 58, 61–69]. In principle, progressing an individual’s physical competence involves developing general, refined and specific movement patterns [64]. One’s capability to apply movement capacities such as ‘balance, coordination, dexterity and hand-eye coordination’ is central for an individual to develop from general to refined and specific movement patterns [64]. As such, movement capacities were evidenced within 44 % of papers in the analysis. Additionally, the importance of developing fine and gross motor skills competence to contribute to one’s physical capacity was evidenced in 18 papers from the analysis [3, 10, 14, 21, 57, 58, 61, 66, 70–79]. FMS were described as a concept that comprises three physical skills, including locomotor, stability and manipulative skills [72]. It was evident that several sport associations use physical literacy and FMS as synonyms, which may represent a departure from the definition of physical literacy advanced by Whitehead [3, 59, 72, 75, 80, 81]. Alternatively, the notion of participating in a range of purposeful physical pursuits literacy was deployed in six papers [11, 18, 66, 71, 82, 83]. According to Almond [83], purposeful physical pursuits “represent a range of activities that can have great significance and value that affect people in a very pervasive manner”. In order to challenge learners’ physical potential and develop their movement patterns, a wide range of physical pursuits should be employed [11, 78, 82] to include physical activity, rhythmic and sport experiences [3, 13, 55]. It is evident that scholars define and interpret ‘physical capabilities’ in several ways. As such, robust empirical research to operationalize the construct is ‘long overdue’ [14].

##### Progression/Developmental Pathway

The following subtheme is concerned with the developmental pathway an individual will progress through in relation to their physical literacy. Three core categories were adopted; namely, throughout the lifespan, unique journey and the Long-Term Athlete Development (LTAD) model. According to seven papers, physical literacy was referred to as a unique journey that individuals experience throughout their lifespan [9, 41, 62, 66, 67, 76, 84]. Although early experiences build the foundations for a lifelong commitment to participate in physical activity [84], there is an emphasis on physical literacy being an important quality throughout the life course [3, 56, 61, 65, 66, 69, 85]. This ‘never-ending’ lifelong journey is also referred to as a ‘cradle to grave’ concept, which is expected to encounter success and setbacks along the way [9, 40]. Every individual has the capacity to develop along their own physical literacy journey, as appropriate to their own capabilities, social and geographical context, and life experiences [40, 58, 62]. Whitehead [55] proposed that individual journeys pass through six different stages according to their age in relation to the development of physical literacy. These stages span (i) preschool; (ii) foundation/early and primary school; (iii) secondary-school years; (iv) early adulthood years; (v) adult years; and (vi) the final stage, older adult years. The LTAD model proposes different physical literacy stages during childhood, adolescence and young adulthood [63], and starts with the active-start stage, which targets 0- to 6-year-olds, passes through a series of stages, and finishes with the train-to-win stage [63].

##### Target Audience

This subtheme is concerned with what the literature deems as the target population to develop physical literacy. Specifically, three core categories were deployed: children, adults, and the notion that all can develop physical literacy. Nineteen papers in the analysis referred to target audiences when discussing physical literacy, and referred to the notion that all individuals are able to develop their physical literacy, including children and adults. According to Taplin [62], all individuals have the capacity to develop their own physical literacy, regardless of their age, ability, weight and height. Thirteen papers defined children and youth populations as a target audience for physical literacy [3, 9, 60, 63, 65, 69, 70, 79, 81, 85–88]. While discussing children, education and PE were frequently associated with physical literacy, with a tendency to view PE as imperative to a child’s education through moving to learn and learning to move [70]. Three papers specifically highlighted the importance of developing physical literacy with the adult population [18, 76, 77]. These papers propose that adults’ physical literacy development can be explained by their previous experiences [18, 76 77]. Adults who continue to progress on their physical literacy tend to be those who have had positive and supportive experiences from significant others and school [18]. On the other hand, the majority of adults who are inactive or occasionally participate in physical activity are those who are more likely to have had negative experiences of physical activity and PE from a young age [18], and therefore tend to have more sporadic physical literacy journeys.

##### Holistic Concept

The holistic subtheme refers to one’s ability to interact with the environment and move with poise and economy. In four papers, developing along one’s physical literacy journey was described as the capacity to communicate with the surrounding environment and society [3, 54, 59, 89]. In a physical sense, 14 papers highlighted that physical literacy is one’s ability to read and interact with all aspects of the physical environment around them [3, 11, 54, 58, 59, 61, 65, 67, 70, 71, 79, 80, 88, 91]. Specifically, an individual progressing along their journey reads the environment “through a range of senses, appreciates, via experience, the relevant components of the display (e.g. shape size, weight, surface, speed, movement of others)” [54]. The richer the interactions with the environment, the greater one will understand their human potential [58]. Another core category of the holistic concept evident in five papers was moving with poise and economy [13, 54, 67, 80, 89]. In line with human’s holistic disposition, one quality of a physically literate individual is demonstrating movement with poise and economy in a range of challenging physical environments [13, 54, 67, 80, 89]. As evidenced above, this holistic subsection predominantly employed Whitehead’s perspective, with eight of a possible 21 papers in the holistic theme written by Whitehead.

##### Related Constructs

The next subtheme evidenced was the related constructs of physical literacy. This subtheme describes constructs that were related to, but not synonymous with, physical literacy. Two core categories were evidenced: health literacy and aesthetic literacy. Four papers made reference to health literacy and aesthetic literacy. Health literacy goes beyond acknowledging and understanding factual health information, but uses the factual information to make an informed decision about one’s health [10, 18, 56]. Alternatively, the concept of aesthetic literacy represented a holistic form of movement that can be perceived via the senses [37]. In this sense, aesthetic literacy resonates with the ‘poise’ aspect of physical literacy identified above and is based on very similar philosophical considerations [37]. However, the discussion surrounding the aesthetic literacy concept focuses primarily on dance and dance education as opposed to all physical pursuits.

#### Philosophical Underpinning for Physical Literacy

Whitehead’s paper [90] on meaningful existence, embodiment and PE has been identified as the philosophical basis for the development of the physical literacy concept. Consequently, a main theme deployed from the papers was the philosophical roots of physical literacy—an interweaving of phenomenology, existentialism and monism. However, one-third of papers (33 %) in the synthesis did not declare or discuss any philosophical considerations. Under the philosophical underpinning higher-order theme, nine core categories were adopted under the following three subthemes: ontological assumptions, epistemological assumptions and pedagogical implications. The structure of the philosophical underpinnings higher-order theme is as follows: (i) ontological assumptions (what is physical literacy from an ontological perspective); (ii) epistemological assumptions (how can physical literacy be studied from an epistemological perspective); and (iii) pedagogical implications (how appropriate pedagogy can influence and help develop physical literacy).

##### Ontological Assumptions

This category denotes the ontological assumptions in relation to the concept of physical literacy. One core category identified is the notion of developing the whole person. Fifteen papers referred to the whole person with reference to Whitehead’s holistic approach to physical literacy, which views human beings as innately holistic [9, 37, 56, 58, 62, 65–67, 69–71, 80, 84, 85, 89]. Three papers referred to holistic education as a means of developing the whole child—attempting to develop mental, physical and emotional attributes that may promote participation in physical pursuits throughout life [9, 70, 79]. Another core category under ontological assumptions identified in sixteen papers was monism and “views a person as essentially an indivisible whole” [40]. According to the papers, a monist philosophical view identifies individuals as an indivisible whole where both mind and body work in unison and are considered equally important [40, 58]. Conversely, a dualist approach views humans as two divisible parts—the mind and the body; often the mind is perceived dominant over bodily capabilities [40, 58, 63, 69, 71, 78, 84].

##### Epistemological Assumptions

This category refers to the epistemological assumptions in relation to the concept of physical literacy, including phenomenology and existentialism. Phenomenology has been described as the way in which an individual perceives the world from their unique point of view, whereas existentialism posits that individuals’ unique perspective arises due to experiences of interacting with the world around us [40, 54, 58]. Eight papers referred to phenomenology and seven papers referred to existentialism [14, 37, 40, 54, 58, 69, 71, 80]. Phenomenology and existentialism are deemed central to the philosophical foundations that formed the physical literacy concept. Specifically, these phenomenological and existential philosophical foundations of physical literacy are fundamental to the interactions between individuals and the environment [75].

##### Pedagogical Implications

The pedagogical implications subtheme contained ten papers and three core categories. Five papers regarded meaningful experiences as a valuable attribute in relation to pedagogical implications [11, 58, 71, 84, 89]. The term ‘meaningful experiences’ encompasses motivating experiences that learners find rewarding and enjoyable, as well as influence their affective domain in developing self-confidence and self-worth [11]. Three papers challenged the pragmatic realities of the philosophical underpinnings of physical literacy [41, 59, 87], while two papers stated that physical literacy is not to be referred to as a pedagogical model [13, 61]. However, it was evident that the ten papers under this subtheme lacked detail on how to practically apply appropriate pedagogy.

#### Associations and Relationship of Physical Literacy

This theme referred to the proposed relationships and causal associations that are claimed for physical literacy, noting that very few of these relationships have been empirically tested to date, although such trials are currently underway. The associations of physical literacy were divided into three subthemes: (i) behavioral characteristics (two core categories); (ii) psychological, social and attitudinal factors (three core categories); and (iii) particular ‘contexts’ where physical literacy can be developed (two core categories).

##### Behavioral Characteristics

This category captured the notion that physical literacy is purported to influence behaviors such as physical activity, health behaviors, sport participation, and an active lifestyle outside of competitive sport. In each case, there are arguments that physical literacy may support these desirable outcomes but also that these behaviors may themselves contribute to enhancing physical literacy. Physical activity was evident in 44 % of the papers and is described as central to the physical literacy concept [72]. Development of the elements of physical literacy (motivation, confidence, physical competence, knowledge and understanding) can lead an individual to participate in physical activity, but, also, to progress one’s physical literacy it is arguably necessary to participate in physical activity. Specifically, the main objective of all physical activity experiences is to ensure individuals develop along their physical literacy journey, and thus have the motivation, confidence, physical competence, knowledge and understanding to value physical activity [11]. Furthermore, the value of physical activity is important in all stages of an individual’s life, with proposed goals at each stage within ones journey [76]; namely, developing motor competence and self-confidence within physical activity was encouraged in the early and primary years, and attention to understanding the importance of physical activity, health and wellbeing throughout the life course was encouraged in secondary schools [13, 76].

Physical literacy can be identified as the basis for the characteristics, attributes, behaviors, awareness, knowledge and understanding towards a healthy lifestyle [59], as well as the foundation to elite sport [86]. Implications that physical literacy improves health were evident in 13 papers. Specifically, six papers referred to obesity, cardiovascular disease and inactivity levels while discussing physical literacy, especially since these factors are more prevalent in recent trends and impact on the health of the nation [21, 65, 70, 78, 86, 87]. This negative trend has implications to a nation in various ways; from an increase in the number of individuals suffering from obesity-related diseases, to a broader financial burden on the healthcare system of the nation [21, 69, 92]. A concurrent message running throughout the 13 papers was how valuing and participating in physical activity was a successful method to impact on health [3, 10, 14, 21, 56, 59, 69, 71, 80, 83, 86, 87, 91]. Therefore, promoting physical literacy will ensure that individuals make healthy and active decisions throughout their life course [3, 10].

##### Psychological, Social and Attitudinal Factors

This subtheme captured the notion that physical literacy is theoretically associated with psychological, social and attitudinal factors such as (a) engagement, enthusiasm and enjoyment during physical activity; (b) support from significant others, and (c) cognitive and academic performance (e.g. in school). In each case, there are arguments that physical literacy may support these desirable outcomes but also that these psychological and attitudinal factors may themselves enhance physical literacy. It was identified that social support from significant others may contribute significantly to the development of physical literacy but, equally, physical literacy may promote the forming of stronger relationships and social networks.

As highlighted in ten papers, significant others play a vital role in promoting learners’ physical literacy [9, 56, 57, 60, 62, 65, 76–78, 86]. Although PE is one means of promoting physical literacy in children and young people, PE teachers are not solely responsible for developing one’s physical literacy [40]. In fact, all significant others, namely “parents, carers, nursery nurses, coaches, peers, family members, leisure management personnel, employers, the medical profession and carers for the elderly” [40], are responsible for impacting and shaping the viewpoints of individuals [62]. Although all significant others have the power to impact positively on one’s physical literacy journey through encouragement, negative comments from significant others can impact negatively on the development of learners’ physical literacy journeys, particularly in children and young people [72, 91].

Four papers reflected the notion of cognitive and academic performance in terms of schooling [61, 65, 69, 87]. It was highlighted that PE in the school system has been regarded for decades as a curriculum subject that contains limited cognitive substance [61]. However, the findings from the analysis suggest that there is growing connection between academic performance and physical fitness [87]. This indicates that being physically active may impact positively on children’s results in school as well as creating a foundation to developing healthy, lifelong habits and physical literacy [87].

##### Contextual Factors

This subtheme captured the notion of particular contexts where physical literacy can be progressed, including (a) PE; and (b) the sport sector. The ‘PE’ subtheme was the most popular core category, with 76 % of papers referring to this concept. A discussion around the relationship between physical literacy and PE was present in a number of papers. A consensus throughout the papers was that developing physical literacy is consistent with the intended outcomes of PE [41, 59, 78, 93]. Specifically, the holistic nature of physical literacy supports the present curricular aims surrounding the development of the whole child, and thus surpasses solely focusing on the physical development of learners [3, 9, 54]. This holistic approach towards PE derives from Whitehead’s philosophical underpinnings for the physical literacy concept [61]. Although PE and sport are connected in many different ways, the goals of these two contextual factors of physical literacy often differ [37]. Eight papers referred to the sport sector as a contextual factor for physical literacy [10, 14, 56, 63, 75, 79, 81, 86]. In sports policies and documents, physical literacy has been described and operationalized by sports organizations mainly in three different forms [10]. The first of these is the discovery of talented athletes and raising sport participation levels [75]; the second is progressing physical attributes such as agility, balance and coordination in an array of physical situations [10]; and the third is for the dual purpose of developing elite athletes as well as encouraging healthy lifelong participation for all [63, 86].

## Discussion

This systematic review has mapped the defining properties, underpinning philosophy and theoretical associations of physical literacy that are reflected in the existing published peer-reviewed literature. Seventy percent of the articles that referred to the term ‘physical literacy’ adopted a ‘Whiteheadian’ perspective. Accordingly, we recommend that researchers be explicit in their definition of physical literacy, the philosophy they adopt and the theoretical predictions they are testing for clarity and consistency. Under philosophy, papers that specified a clear philosophical standpoint focused on the ‘Whiteheadian’ combination of phenomenology, existentialism and monism. The following discussion considers whether this is the only philosophical approach to physical literacy, and the implications of adopting different philosophical assumptions. Regarding theoretical associations, several broad categories of associations are identified in the existing literature, although the directions of these relationships are rarely made clear in the papers sampled. The following discussion explores whether (a) this is an exhaustive and complete list of theoretical associations; and (b) how the predictions detailed in this analysis might be tested and evaluated appropriately.

### Defining Properties

Overall, common themes from the data highlight that physical literacy is conceptualized as the interactive and simultaneous consideration of competence in physical skills, confidence, motivation towards physical pursuits, and the valuing of physical movement and/or interacting with the physical world (see, for example, Whitehead [40] and Taplin [62]). The concept is applicable across the lifespan, to individuals of all ability levels, and will be experienced differently by every person, resulting in an individual ‘physical literacy journey’. Physical literacy differs from related constructs such as health literacy and aesthetic literacy. Within the analysis, there are tendencies towards two recognizable approaches, or traditions, to physical literacy: a ‘Whiteheadian’ approach [13], and the ‘LTAD’ approach [94], with the latter appearing to focus more on developing physical literacy through, and for, sport participation. Overall, reflecting the arguments of Whitehead (eight papers) and Almond (six papers), the analysis suggests that the Whiteheadian conceptualization of physical literacy covers a wider range of movement types/skills, and psychosocial attributes, as it extends beyond competitive sport participation as the main vehicle for ‘purposeful physical movements’. On the other hand, in all of the papers discussing the LTAD paradigm, the LTAD focuses on developing the physical elements of physical literacy [3, 59, 63, 75, 86]. As such, it might be argued that the LTAD model can be accommodated within the Whitehead model, but not vice versa. However, more recent LTAD literature suggests that the LTAD is not just unique to sport participation, but is also relevant to PE, recreation, and free-play environments [94].

The analysis presented in this paper reflects a unique synthesis drawing from a wide range of sources, and also reflecting the consistency and prevalence of key themes in papers that directly relate to ‘physical literacy’. Reflecting on the nature of the findings generated through the above approach, most of the papers that seek or achieve publication in peer-reviewed academic journals adopt a conceptualization based on the ‘Whiteheadian’ definition [13]. While our analysis reflects aspects of different approaches to physical literacy, 70 % of the papers in this study (35 papers) adopted the conceptualization put forward by Whitehead [13], of which eight papers were written by Whitehead herself. Our analysis highlights key differences between different standpoints; namely, inconsistencies between a holistic definition and a definition solely from the physical domain. A necessity to either resolve these differences or accept and embrace diverse approaches to promoting physical literacy is pertinent. For example, different cultures, governance structures, geographical locations and physical environments may necessitate different conceptualizations and pedagogies for physical literacy.

### Philosophical Underpinnings

Our analysis sought to identify the philosophical underpinnings of physical literacy in terms of (a) the aims and purpose of physical literacy; (b) the ontology and epistemology of physical literacy; and (c) the pedagogy of promoting and supporting physical literacy. This assumption proposes that one’s experience and interpretation of the world is heavily dependent on one’s ability to perceive physical cues, and respond meaningfully in the physical realm. These aims are aligned to the ontological and epistemological assumptions that arise from attempting to combine phenomenology, existentialism and monism. Such a combination of assumption sets is challenging for practitioners and researchers to access, operationalize and put into practice. Therefore, these assumptions require further articulation or better communication in order to connect with both researchers and practitioners working in this area. Once these aims and underlying assumptions are accepted, then the resulting pedagogy must focus on the whole person, the individualized learning, ipsative evaluation that focuses on individual progression, and contextualized real-world experiences (i.e. not simulated, abstract training, such as drills) [16]. Notably, this was the only philosophy offered by the papers included in our analysis, with no alternative approaches to philosophical underpinnings available. It may become important to consider how physical literacy would be operationalized under different assumption sets, such as empiricism, post-positivism, and critical realism. For example, does physical literacy theory lend itself to objective testing of effectiveness?

Furthermore, in line with Whitehead’s ipsative, individualized assessment [16], one potential problem is the interpretation of standards and assessment within physical literacy. In addition, given the relative paucity of philosophical consideration in many scientific papers, does physical literacy lend itself to being studied and tested by those unfamiliar with philosophical assumptions? Failing that, can we expect each researcher or teacher/practitioner to engage with detailed ontology and philosophy prior to engaging with this concept? While philosophy may be quite an unpopular and impenetrable topic, resolving some of the above issues may genuinely energize both the scientific study and practical delivery of physical literacy.

Nonetheless, the philosophical underpinnings and the properties of physical literacy seem to be ill-aligned in research to date, specifically the predominant philosophy of monism, meaning that mind and body are one indivisible whole [40]. However, the physical, psychological and behavioral properties in physical literacy are largely considered as separate entities, although clearly interlinking constructs. As such, the question of how to promote the relative importance of each factor is left unconsidered in the majority of physical literacy research to date.

### Theoretical Associations and Relationships

Our analysis sought to identify the concepts and constructs that are frequently linked to physical literacy, as understanding the proposed determinants and outcomes of physical literacy allows for the development and testing of specific hypotheses. Furthermore, if supported, these relationships would form the core justification for practitioners choosing to adopt a physical literacy approach, or to choose physical literacy over other existing approaches. Our findings suggest that physical literacy is proposed to be associated with a wide array of behavioral, psychological, social and physical variables, as well as linked to specific contexts in which physical literacy can be developed. It is unlikely that the list generated in our analysis is exhaustive, as elsewhere physical literacy has been linked to outcomes such as cardiovascular fitness, strength, motor skill, and obesity/overweight status [17]. Hence, our analysis may simply reflect the most accepted and salient associations made with physical literacy. Additionally, however, it was extremely rare for papers to specify the direction of the relationship between physical literacy and its associated construct, and bidirectional causation was plausible in many cases. As such, there is an emerging need to both test which variables contribute to the development of physical literacy and test those that are enhanced by the development of physical literacy. As noted above, the nature of the experiments and tests used should, ideally, be aligned to a specific philosophical approach, and in many ways the approach offered by combining phenomenology, existentialism and monism does not submit as readily to traditional empirical testing, such as randomized controlled trials. In this case, the research community will need to debate and agree what would count as sufficient evidence to claim efficacy within specific philosophical assumption sets.

### Limitations

The limitations of this systematic review include (i) only papers in the English language were considered, thus the papers were primarily derived from the UK and Canada; and (ii) no empirical data or measurement attempts for physical literacy were considered for this systematic review as the authors’ aim and research questions were to discuss the properties of physical literacy, philosophical underpinnings and its associations. Nevertheless, a review of empirical data and attempts to measure physical literacy needs to be addressed in future research papers. Furthermore, this systematic review does not answer the question of which definition and philosophy is correct, which may be viewed as a limitation. However, the review recorded and discussed the various definitions and philosophies, which was the most appropriate course of action.

## Conclusions

This paper is the first to provide a systematic review of core attributes of the physical literacy construct, including the defining properties of physical literacy, the philosophical foundations and the theoretical associations of the construct. As identified, there have been many references to the physical literacy theory and its implications to practice. Five databases were searched using the PRISMA guidelines for systematic reviews. Inclusion criteria were English language, peer reviewed, published by March 2016, and seeking to conceptualize physical literacy. A total of 50 published articles met the inclusion criteria and were analyzed qualitatively using inductive thematic analysis. Three higher-order themes were adopted from the thematic analysis on the 50 papers selected following the rigorous PRISMA guidelines; namely, qualities of physical literacy, philosophical underpinning for physical literacy, and the connection between physical literacy and PE. This paper has illustrated the importance of adopting a pragmatic perspective to physical literacy but acknowledges that physical literacy represents more than solely a physical concept.

An implication for theory development and research is the need for transparency and tolerance with different approaches to physical literacy. The authors acknowledge the philosophical perspective but recognize a more pragmatic perspective reflecting the evidence-based society that is lived within to track whether individuals are making progress along their physical literacy journey. This approach would enable researchers to operationalize the construct of physical literacy and establish meaningful, measureable differences. Implications for applied practice include ensuring clarity of theoretical descriptions and phrases so that these can be translated clearly into a practical setting. For example, ‘reading the environment’ can be misinterpreted in many ways, thus making it difficult for practitioners to understand what this concept looks like in practice. Additionally, it is pertinent for practitioners to consider that there are other factors that disengage individuals from taking part in physical activity throughout the life course, such as the fear of being discriminated against by others [41]. It has been established that physical literacy and FMS are not synonyms; FMS focuses on progressing physical skills only, whereas physical literacy also considers the affective and cognitive elements [91]. One implication of this is that FMS may play a role in a broader program of physical literacy as a way of developing the physical competence element of physical literacy. This implication indicates that if the locomotor, stability and manipulative strands of FMS are completed in an applied setting, this could be one method, alongside others, to help develop the physical competence aspect of physical literacy.

Recommendations for future research include not only the need for transparency and clarity but also tolerance of different approaches, providing researchers are transparent and clear in what they did/assumed. Consequently, this would lead to a pluralism where different ideas can compete and be evaluated over time, without which it would be impossible to decipher whether physical literacy is being tested, supported or refuted. Ultimately, researchers need to operationalize physical literacy and generate meaningful, measurable differences that will eventually be the arbiter of what physical literacy is and how it works.

## Electronic supplementary material

Below is the link to the electronic supplementary material.

Supplementary material 1 (DOCX 653 kb)

Supplementary material 2 (DOCX 19 kb)

Supplementary material 3 (DOCX 41 kb)

## References

[1] Delaney B, Donnelly P, News J (2008). Improving physical literacy.

[2] Higgs C, Balyi I, Way R (2008). Developing physical literacy: a guide for parents of children ages 0 to 12.

[3] Mandigo J, Francis N, Lodewyk K (2009). Physical literacy for educators. Phys Health Educ..

[4] Keegan RJ, Keegan SL, Daley S (2013). Getting Australia moving: establishing a physically literate and active nation (game plan).

[5] Youth Sport Trust (2013). Primary school physical literacy framework.

[6] Keegan RJ, Edwards L, Bryant A, et al. A systematic review of the definitions, foundations and associations of physical literacy. Australian Conference of Science and Medicine in Sport: Sanctuary Cove; 21–24 Oct 2015.

[7] Schools and Physical Activity Task and Finish Group (2013). Physical literacy—an all-Wales approach to increasing levels of physical activity for children and young people.

[8] Tremblay M (2012). Major initiatives related to childhood obesity and physical inactivity in Canada: the year in review. Can J Public Health.

[9] Liedl R (2013). A holistic approach to supporting physical literacy. Phys Health Educ..

[10] Macdonald D, Enright E. Physical literacy and the Australian health and physical education curriculum. ICSSPE Bull J Sport Sci Phys Educ. 2013;65:351–59.

[11] Whitehead M, Almond L. Creating learning experiences to foster physical literacy. ICSSPE Bull J Sport Sci Phys Educ. 2013;65:72–9.

[12] World Health Organization. Obesity and overweight. 2015. Available at: http://www.who.int/mediacentre/factsheets/fs311/en/. Accessed 12 Feb 2015.

[13] Whitehead M. Definition of physical literacy and clarification of related. ICSSPE Bull J Sport Sci Phys Educ. 2013;65:28–33.

[14] Giblin S, Collins D, Button C (2014). Physical literacy: importance, assessment and future directions. Sports Med.

[15] Warburton D, Nicol C, Bredin S (2006). Health benefits of physical activity: the evidence. CMAJ.

[16] Whitehead M (2010). Physical literacy: throughout the lifecourse.

[17] Gately P, Whitehead M (2010). Physical literacy and obesity. Physical literacy: throughout the lifecourse.

[18] Almond L. What is the relevance of physical literacy for adults? ICSSPE Bull J Sport Sci Phys Educ. 2013;65:214–22.

[19] Wang F, McDonald T, Reffitt B (2005). BMI, physical activity, and health care utilization/costs among Medicare retirees. Obes Res.

[20] British Heart Foundation National Centre (2013). Economic cost of physical inactivity: making physical activity a priority.

[21] Moreno T. American physical education: a discursive essay on the potential unifying role of physical literacy in the United States. ICSSPE Bull J Sport Sci Phys Educ. 2013;65:371–77.

[22] Alagul O, Gursel F, Keske G (2012). Dance unit with physical literacy. Proc Soc Behav Sci..

[23] Guo Y, Justice L, Kaderavek J (2012). The literacy environment of preschool classrooms: contributions to children’s emergent literacy growth. J Res Read..

[24] Macdonald D, Abbott R, Hunter L (2014). Physical activity—academic achievement: student and teacher perspectives on the ‘new’ nexus. Phys Educ Sport Pedag..

[25] Caput-Jogunica R, Lončariž D, De Privitellio S (2009). Extracurricular sports activities in preschool children: impact on motor achievements and physical literacy. Croat Sports Med J..

[26] Liebenson C (2009). Training for speed. J Bodyw Mov Ther..

[27] Sharpe E, Forrester S, Mandigo J (2011). Engaging community providers to create more active after-school environments: results from the Ontario CATCH kids club implementation project. J Phys Act Health..

[28] Sheehan D, Katz L (2011). The pursuit of physical literacy: can exergaming develop fundamental movement skills like balance?. Can J Diabetes..

[29] Sheehan D, Katz L (2013). The effects of a daily, 6-week exergaming curriculum on balance in fourth grade children. J Sport Health Sci..

[30] Haughey T, Breslin G, Toole S, et al. Developing physical literacy through coach education: a Northern Ireland perspective. ICSSPE Bull J Sport Sci Phys Educ. 2013;65:252–56.

[31] MacCallum M, Sheehan D (2013). Kids in kilts: using highland dance to develop fundamental movement skill. Phys Health Educ..

[32] McKee M, Breslin G, Haughey T, et al. Research into assessing physical literacy in Northern Ireland. ICSSPE Bull J Sport Sci Phys Educ. 2013;65:283–88.

[33] McKee M, Breslin G, Haughey T, et al. Physical literacy co-ordinators and active school partnerships in Northern Ireland. ICSSPE Bull J Sport Sci Phys Educ. 2013;65:299–305.

[34] Sun H (2013). Impact of exergames on physical activity and motivation in elementary school students: a follow-up study. J Sport Health Sci..

[35] Reynolds J, Thornton A, Lay B (2014). Does movement proficiency impact on exergaming performance?. Hum Mov Sci.

[36] Kentel J, Dobson T (2007). Beyond myopic visions of education: revisiting movement literacy. Phys Educ Sport Pedag..

[37] Lussier C (2010). Aesthetic literacy: the gold medal standard of learning excellence in dance. Phys Health Educ..

[38] Mandigo J, Holt N (2004). Reading the game: introducing the notion of games literacy. Phys Health Educ..

[39] Lakatos I, Lakatos I, Musgrave A (1970). Falsification and the methodology of scientific research programmes. Criticism and the Growth of Knowledge.

[40] Whitehead M. The history and development of physical literacy. ICSSPE Bull J Sport Sci Phys Educ. 2013;65:21–7.

[41] Hylton K. Physical literacy, ‘race’ and the sociological imagination. ICSSPE Bull J Sport Sci Phys Educ. 2013;65:223–27.

[42] Khan KS, Kunz R, Kleijnen J (2003). Systematic reviews to support evidence-based medicine: how to apply findings of healthcare research.

[43] Higgins J, Green S. Cochrane handbook for systematic reviews of interventions. Version 5.1.0 [updated March 2011]. The Cochrane Collaboration; 2011. Available from: http://www.cochrane-handbook.org.

[44] Evans D. Appraising systematic reviews. Chang Pract. 2000; Suppl:1–6.

[45] Schlosser RW (2007). Appraising the quality of systematic reviews. Focus..

[46] Moher D, Liberati A, Tetzlaff J (2009). Preferred reporting items for systematic reviews and meta-analyses: the PRISMA statement. PLoS Med..

[47] Ahmed I, Sutton AJ, Riley RD (2012). Assessment of publication bias, selection bias, and unavailable data in meta-analyses using individual participant data: a database survey. BMJ.

[48] Gibbs GR (2007). Analyzing qualitative data.

[49] Guest G, MacQueen K, Namey E (2012). Applied thematic analysis.

[50] Hay I (2005). Qualitative research methods in human geography.

[51] Wilson DB, Cooper H, Hedges LV, Valentine JC (2009). Systematic coding. The handbook of research synthesis and meta-analysis.

[52] Braun V, Clarke V (2006). Using thematic analysis in psychology. Qual Res Psychol..

[53] Boyatzis RE (1998). Transforming qualitative information.

[54] Whitehead M (2001). The concept of physical literacy. Br J Teach Phys Educ..

[55] Whitehead M. The value of physical literacy. ICSSPE Bull J Sport Sci Phys Educ. 2013;65:41–42.

[56] Roetert EP, Jefferies SC (2014). Embracing physical literacy. J Phys Educ Recreat Dance..

[57] Silverman S, Mercier K (2015). Teaching for physical literacy: implications to instructional design and PETE. J Sport Health Sci..

[58] Whitehead M (2007). Physical literacy: philosophical considerations in relation to developing a sense of self, universality and propositional knowledge. Sport Ethics Philos..

[59] Corlett J, Mandigo J (2013). A day in the life: teaching physical literacy. Phys Health Educ..

[60] Ennis CD (2015). Knowledge, transfer, and innovation in physical literacy curricula. J Sport Health Sci..

[61] Kirk D (2013). Educational value and models-based practice in physical education. Educ Philos Theory..

[62] Taplin L (2011). Physical literacy: an introduction to the concept. Phys Educ Matters..

[63] McCaffery M, Singleton E (2013). Why are we doing this anyway? Physical literacy, monism, and perceived physical competence for Ontario’s elementary students. Phys Health Educ..

[64] Whitehead M. Content implications of working to promote physical literacy. ICSSPE Bull J Sport Sci Phys Educ. 2013;65:89–97.

[65] Castelli DM, Centeio EE, Beighle AE (2014). Physical literacy and comprehensive school physical activity programs. Prev Med.

[66] Dudley DA (2015). A conceptual model of observed physical literacy. Phys Educ.

[67] Hastie PA, Wallhead TL (2015). Operationalizing physical literacy through sport education. J Sport Health Sci..

[68] Lounsbery M, McKenzie TL (2015). Physically literate and physically educated: a rose by any other name?. J Sport Health Sci..

[69] Sprake A, Walker S (2015). ‘Blurred lines’: the duty of physical education to establish a unified rationale. Eur Phy Educ Rev..

[70] Marsden E, Weston C (2007). Locating quality physical education in early years pedagogy. Sport Educ Soc..

[71] Gallant P, Vossen D, Weaving C (2011). In the zone: physical literacy and the quest for certified coaches. Phys Health Educ..

[72] Almond L. Physical literacy and fundamental movement skills: an introductory critique. ICSSPE Bull J Sport Sci Phys Educ. 2013;65:80–88.

[73] Flemons M. The reconceptualisation of gymnastics: equipping physical education teachers to promote physical literacy in schools. ICSSPE Bull J Sport Sci Phys Educ. 2013;65:189–198.

[74] López de D’Amico R. About physical literacy in Venezuela. ICSSPE Bull J Sport Sci Phys Educ. 2013;65:367–70.

[75] Pot N, Hilvoorde I. A critical consideration of the use of physical literacy in the Netherlands. ICSSPE Bull J Sport Sci Phys Educ. 2013;65:312–19.

[76] Whitehead M. Stages in physical literacy journey. ICSSPE Bull J Sport Sci Phys Educ. 2013;65:51–55.

[77] MacDonald LC (2015). Moving high school students toward physical literacy. J Phys Educ Recreat Dance..

[78] Roetert ER, MacDonald LC (2015). Unpacking the physical literacy concept for K-12 physical education: what should we expect the learner to master?. J Sport Health Sci..

[79] Corbin CB (2016). Implications of physical literacy for research and practice: a commentary (review). Res Q Exerc Sport.

[80] Jurbala P (2015). What is physical literacy, really?. Quest..

[81] Lundvall S (2015). Physical literacy in the field of physical education—a challenge and a possibility. J Sport Health Sci..

[82] Almond L. What is the value of physical literacy and why is physical literacy valuable? ICSSPE Bull J Sport Sci Phys Educ. 2013;65:34–40.

[83] Almond L. Physical literacy and its association with health. ICSSPE Bull J Sport Sci Phys Educ. 2013;65:228–35.

[84] Sprake A, Walker S. “Strike while the iron is hot”: the duty of physical education to capitalise on its compulsory position with a holistic curriculum underpinned by physical literacy. ICSSPE Bull J Sport Sci Phys Educ. 2013;65:43–50.

[85] Sun H (2015). Operationalizing physical literacy: the potential of active video games. J Sport Health Sci..

[86] Mandigo J, Harber V, Higgs C, et al. Physical literacy within the educational context in Canada. ICSSPE Bull J Sport Sci Phys Educ. 2013;65:360–66.

[87] Weiler R, Allardyce S, Whyte G (2014). Is the lack of physical activity strategy for children complicit mass child neglect?. Br J Sports Med.

[88] Chen A (2015). Operationalizing physical literacy for learners: embodying the motivation to move. J Sport Health Sci..

[89] Petherick L (2013). “Enlivening” the quality school intramural recreation program advisory committee: revisiting the philosophical and social spaces of intramural and recreation extra-curricular activities. Phys Health Educ..

[90] Whitehead M (1990). Meaningful existence, embodiment and physical education. J Philos Educ..

[91] Almond L. Translating physical literacy into practical steps: the role of pedagogy. ICSSPE Bull J Sport Sci Phys Educ. 2013;65:63–71.

[92] Moreno T (2011). Physical education is affordable healthcare. Strategies..

[93] Penney D, Chandler T (2000). A curriculum with connections?. Br J Teach Phys Educ..

[94] Balyi I, Way R, Higgs C (2013). Long-term athlete development.

