# Supplementary material for: Definitions, Foundations and Associations of Physical Literacy: A Systematic Review
Source: Sports Med. 2016 Jun 30;47(1):113–26. doi: 10.1007/s40279-016-0560-7 (PMC5215133; doi:10.1007/s40279-016-0560-7)
Supplement: Supplementary file 3 — Supplementary material 3 (DOCX 41 kb) [file 40279_2016_560_MOESM3_ESM.docx]

Electronic Supplementary Material Appendix S3

Table of authors, year, focus of study, key words and phrases for the thematic analysis and journal.

| Authors | Year | Focus of the study | Key words/ phrases for thematic analysis | Journal |
| --- | --- | --- | --- | --- |
| Penney & Chandler | 2000 | “In this article we seek to extend debates about these issues (changes in the National Curriculum of Physical Education) and specifically, add new level of detail to our discussion of possible ‘alternatives’.” | PE, National Curriculum, movement and PL, variety of physical activities, engagement, individual development, PE to develop social/ psychological, knowledge and understanding, bodily awareness, enjoyment, participation. | British Journal Of Teaching Physical Education |
| Whitehead | 2001 | “The intention of this paper is to open a debate on the concept of Physical Literacy. This is a term that has been used intermittently over the years but has never received serious analysis” | PE, NCPE/ initial teacher training, 1993 Whitehead introduced concept, embodied capacities – importance, literacy justification, philosophical foundations, monism versus dualism, existentialists and phenomenologists, interacting with surroundings – richer the interactions, read the environment, universal concept – all individuals interact, physically challenged – disabled – PL relative to their embodied dimension, movement capacities – balance, coordination etc., move with poise & economy, confidence, development of self- confidence, self-esteem. | British Journal of Teaching **Physical** Education |
| Whitehead | 2007 | “This paper opens with a presentation of the philosophical underpinning and rationale of the concept of physical literacy. This is followed by an articulation of the concept of physical literacy.” | Reject dualism, monist/holistic view, embodied life as we know it, mind and body as one, interaction with environment, play vital role in human life, Whitehead, motivation, confidence, knowledge and understanding, physical competence, movement capacities, motor ability, achievable by all, self- esteem reading aspects of physical environment, sense of self, phenomenological/existentialist perspective, universality of PL, PE, sport in school, personally rewarding experience. | Sport, Ethics & Philosophy |
| Marsden & Weston | 2007 | “This paper examines the apparent mis-match between the needs of children in their early years of compulsory  schooling and the present philosophy of sports based physical education” | Early years pedagogy, inactivity/ obesity epidemic, holistic PE, child – centred, read physical environment, intrinsic motivation, quality PE, social, emotional, developmental needs of children, media on participation, motor ability, Whitehead, explore / interact, whole child. | Sport, Education & Society |
| Mandigo, [Francis,](javascript:__doLinkPostBack('','ss~~AR%20%22Francis%2C%20Nancy%22%7C%7Csl~~rl','');) [Lodewyk,](javascript:__doLinkPostBack('','ss~~AR%20%22Lodewyk%2C%20Ken%22%7C%7Csl~~rl','');)  & [Lopez](javascript:__doLinkPostBack('','ss~~AR%20%22Lopez%2C%20Ron%22%7C%7Csl~~rl','');) | 2009 | “The following summary provides background information on physical literacy through the lens of an educator. The paper concludes with a proposed working definition of physical literacy for consideration by educators across Canada.” | Literacy broader than reading/writing, social practices and relationship, Long Term Athlete Development (LTAD) model, creative and critical skills, move confidently, FMS, wide range of physical activities, movement competence, ‘read’ environment, development physical skills, motor ability, PE, children develop skills, knowledge, physical competence, lead to healthy lifestyle, affective characteristics, motivation, confidence, Whitehead, throughout lifespan, health- related. | Physical & Health Education Journal |
| Lussier | 2010 | “The purpose of this paper is thus to explore the notion of aesthetic literacy in dance, to discuss where and how it is similar and expands on our notions of physical literacy, and finally, to elaborate on how we can apply this notion of aesthetic, physical literacy into our dance classes.” | Ability to “read” human body – body language, athletes – embodied experience, literate in movement language, dance, aesthetic literacy similar to PL, PE, Mandigo *et al*. (2009) reference, values whole person, phenomenological – engagement in activities, move with competence, complementary perspective to PL, pedagogical implications, imaginative learning. | Physical & Health Education |
| [Taplin](javascript:__doLinkPostBack('','ss~~AR%20%22Taplin%2C%20Liz%22%7C%7Csl~~rl','');) | 2011 | “This article aims to explain the [physical literacy] concept and to persuade colleagues that physical literacy is not just another initiative” | PE, holistic nature of PL, Whitehead definition, PA throughout life course, cradle to grave concept, all can develop PL, elements of PL, motivation, confidence, knowledge and understanding, physical competence, PL journey, PL goal of PE, significant others have positive and negative effect. | [Physical Education Matters](javascript:__doLinkPostBack('','mdb~~s3h%7C%7Cjdb~~s3hjnh%7C%7Css~~JN%20%22Physical%20Education%20Matters%22%7C%7Csl~~jh','');) |
| Gallant, Vossen & [Weaving](javascript:__doLinkPostBack('','ss~~AR%20%22Weaving%2C%20Charlene%22%7C%7Csl~~rl','');) | 2011 | “After detailing the scope of the coaching problem, we enhance the existential foundation for physical literacy that Whitehead provides, adding a more detailed analysis of the philosophical assumptions underlying the historical denigration of sport and coaches. Our intent is to champion Whitehead's existential platform and to highlight the essential influence of properly certified coaches within this realm of embodied experience.” | Whitehead, holistic approach, understand environment, embodied conception, previous experiences, meaningful experiences, philosophical foundation, purposeful interactions, coaching development, existentially enriching environment, dualistic – bodily capacities less important, promote moral character, movement capacity, physical mental health, moral, existential, phenomenological perspective, motor ability. | Physical & Health Education Journal |
| Moreno | 2011 | “The introduction of physical literacy as a concept may be an important tool to help physical educators in the US unify in approach in enabling youth and future generations to appreciate physical activity, reduce the prevalence of obesity, and decrease health care costs.” | PE is affordable healthcare, schools to encourage youth they are responsible for their health and wellbeing, health insurance, movement literacy, promote PL campaign, learn competently and confidently move. | Strategies: A Journal for Physical and Sport Educators |
| Almond | 2013 a | “The starting point for this article is to pose two questions (1) what is the value of physical literacy? and (2) why is it valuable? In this article I propose to answer these questions in order to illuminate the significance of physical literacy and put them in the context of what there is to understand.” | Value of PL, value on personal and professional levels, enhance quality of life, participation influence wellbeing, purposeful physical pursuits, understanding PL, beyond just engagement – enthuse. | International Council of Sport Science and Physical Education (ICSSPE Bulletin Paper) |
| Almond | 2013 b | “In this article I shall identify some problems with the association of physical literacy with fundamental movement skills (FMS)...My purpose in doing this is to raise awareness of the complexity of an association between FMS with physical literacy...” | FMS – problems/ misunderstanding, debate on FMS/PL, definitions including FMS, motor ability, PE, examples from Canada, New Zealand, Australia., FMS is locomotor, body management, manipulative, movement competence, importance of motivation and confidence. | ICSSPE Bulletin Paper |
| Almond | 2013 c | “In this article I propose to explore how physical literacy can be applied to adults. I shall raise questions about the process in which practitioners work with adults. This will lead to a discussion on empowerment and its significance in the promotion of physical literacy.” | Whitehead, value and take responsibility, significance for adults, purposeful physical pursuits, role of practitioners, current physical activity guidelines, inactivity levels. | ICSSPE Bulletin Paper |
| Almond | 2013 d | “In this article I propose first to explore the relationship between purposeful physical pursuits and health benefits, and secondly, the relevance of recent discussions on health literacy to physical literacy.” | Relationship between PL and health, health literacy, physical activity promotion, personal responsibility. | ICSSPE Bulletin Paper |
| Almond | 2013 e | “This paper discusses a number of pedagogical implications of working to promote physical literacy. It looks at a pedagogy for translating physical literacy into action steps, a pedagogical process, pedagogical skills, relational pedagogy and the importance of giving learners a voice. The paper challenges practitioners to reflect on their work with learners and develop a pedagogy of engagement.” | PL complex and simple, variety physical pursuits, translate to practical steps, reaching out to learners that are unmotivated, reluctant, disadvantaged., confidence, pedagogical process, positive environment, practitioners must engage, enthusiastic, empathetic, supportive environment. | ICSSPE Bulletin Paper |
| Corlett & Mandigo | 2013 | “In the world of physical education and sport, the word "literacy" has been adapted to purposes beyond its birthplace in the world of language. The idea of physical literacy has become part of the discourse among physical educators and also those with an interest in athlete -development” | Meaning of ‘literacy’, more than read or write, PE, literacy in physical sense, knowing more than physical movements, develop whole person, read environment, behaviours to a healthy lifestyle, movement competence, knowledge, confidence, motivation, self-competence, movement begins at birth, pragmatic reality of PL, LTAD model, comparison to maths, music, English, FMS- building blocks, physical alphabet. | Physical & Health Education Journal |
| Flemons | 2013 | “This paper considers the way in which Gymnastics has been marginalised in the physical education curriculum and proposes a review of teaching methods which would benefit Gymnastics and enable the activity to play a part in promoting physical literacy.” | Gymnastics in PE, positive effect on physical, social, cognitive, affective, teacher’s beliefs – lack of understanding PL, lack of gymnastics being taught, motor ability, Whitehead definition, beliefs lead to barriers when teaching, creativity. | ICSSPE Bulletin Paper |
| Hylton | 2013 | “This paper argues that in relation to physical literacy we must continue to problematise the philosophies that underpin the concept to the point that it is less utopian, idealistic and politically neutral.” | Philosophies of PL to become less utopian, PL journey, less theoretical and more approachable, pragmatic and realist approach, PE, physically illiterate. | ICSSPE Bulletin Paper |
| Kirk | 2013 | “My purpose in this paper is to revisit the enduring conundrum of physical education’s situation in the school curriculum and to offer a different way of thinking about educational value from what I will call a models-based approach.” | PE, sport education models, PE’s lack of credibility (sport in 80’s), Whitehead, interact with environment, motivation, knowledge and understanding, throughout life course, PL not a pedagogical model, motor ability, nature of schools is ‘cognitive content’, monism, confidence, physical competence, movement capacity, disposition to capitalise on innate movement. | Educational Philosophy & Theory |
| Liedl | 2013 | “... children to grow up to be well-adjusted and caring individuals, who will have the skills, knowledge, confidence, and motivation to be contributing members of society. Parents want the total package for their children, and a holistic approach to education, in which the mental, physical, and emotional domains of the student are considered.” | Knowledge, confident, motivated, children, parents perspective – total package, holistic approach to education, mental, physical, emotional, lifelong journey, PE, quality PE, develop whole child. | Physical & Health Education Journal |
| López de D’Amico | 2013 | “This essay presents a short view of how the term physical literacy is perceived in Venezuela, a country in which this term has not been used, nonetheless it seems that there have been elements in place that look at the objectives of physical literacy. So in order to present the information there is a review of the historical development of Venezuelan society in connection with its evolution and later its education, mostly physical education.” | PL in Venezuela, PL not establishes in Venezuela, PE, physical activity promotion, importance of understanding PL properly, motor skills, reference to indigenous people – no difference between work and exercise. | ICSSPE Bulletin Paper |
| Macdonald & Enright | 2013 | “The HPE curriculum does not make explicit reference to the concept of physical literacy... There are, however, strong alignments between particular interpretations of physical literacy and the HPE curriculum. In future iterations of HPE we see possibilities for physical literacy to be included as a “general capability” reflecting the interpretations of physical literacy that describe it as a human or embodied capability.” | PL in Australia, health and PE curriculum, health literacy, health promotion, PL cornerstone to lifelong participation, Whitehead, movement competence, affective and cognitive domains, motor skills, sport sector. | ICSSPE Bulletin Paper |
| Mandigo, Harber, Higgs, Kriellaars &  Way | 2013 | “This paper looks at the development of physical literacy in Canada over the last 10 years. The discussion is based round some of the strategies to promote physical literacy, as identified by Margaret Whitehead (2010)*.”* | PL in Canada, children sedentary behaviour – spending time on computers, PL through grassroots sports, education, sport, recreation and public health, holistic and monistic, PL inclusive of all, children, movement skills, Canadian curriculum, LTAD framework, coaching, parents. | ICSSPE Bulletin Paper |
| McCaffery & Singleton | 2013 | “The purpose of this article to first explore three meanings attached to the concept of physical literacy: (a) that proposed by Margaret Whitehead who created the term; (b) the description provided by the Canadian Sport for Life movement; and (c) the definition stated in the Ontario Curriculum, Grades 1-8:Health and Physical Education {Interim Edition) (2010).” | Personal embodiment, unique and individual, throughout life course, disembodied and dualistic, monism, physical competence – making progress as oppose to achieving mastery, interact with the world, LTAD (Canada), movement skills/ competencies, perceived competence – factor enjoyment and participation, children, motivation, some monism lost in translation, different interpretations, physical activity. | **Physical** & Health Education Journal |
| Moreno | 2013 | “The introduction of physical literacy as a concept may be an important tool to help physical educators in the US unify in approach in enabling youth and future generations to appreciate physical activity, reduce the prevalence of obesity, and decrease health care costs.” | US perspective – evolving concept, PE preventative approach to inactivity levels, motor skills, movement competence, PE is affordable healthcare, healthcare and economy, obesity related diseases, fitness programme concerns, Whitehead, three PL elements. | ICSSPE Bulletin Paper |
| Petherick | 2013 | “My purpose in writing this note is not to suggest radical change is necessary (although sometimes this might be the best option!) but rather to "enliven" the possibility for doing things a little different, engaging diverse and different students in the planning process, or tapping into unused resources or strengthening community connections to foster school culture, student engagement, and student learning.” | Whitehead, move with poise and economy, positive experiences, achieve personal success, engagement, participation various activities, gathering interest, collections as oppose to individualism in intramural, holistic, social aspects, intramurals depends on teaching and likeminded people, confident and competent movers, likely to leadership qualities. | Physical and Health Education |
| Pot & Hilvoorde | 2013 | “First, based on the philosophical foundations of physical literacy, it can be argued that movements have a different meaning in different contexts. Second, the idea that learning fundamental movements will lead to sport participation is sociologically problematic. Third, the focus on elite athleticism does not seem to be in line with the basic principles of physical literacy.” | PL in the Netherlands, PL and FMS are used an synonyms, term used in Sport Associations, focus on elitism, Whitehead, difference between PL and FMS, reference to LTAD model, motor internationality, active lifestyle versus elite sports, PL translates to physical alphabet in the Netherlands. | ICSSPE Bulletin Paper |
| Sprake & Walker | 2013 | “This paper explores the philosophical controversies surrounding Physical Education within a curriculum shackled to a dualist perspective on what is educationally valuable.” | PE – positive experience, holistic education, current society/ education is dualist – intellectual development is priority, PL journey, movement capacity, monist outlook on human embodiment, development of a disposition. | ICSSPE Bulletin Paper |
| Whitehead | 2013 a | “This article sets out the current definition of physical literacy and then discusses a range of issues, many of which seek to clarify the concept and counter misinterpretations that have dogged the development and acceptance of the concept” | Each individuals endowment, human disposition, motivation, confidence, movement competence, knowledge and understanding, physical competence, monism, relationship between PL and PE, PL – encapsulating value of physical activity as an end in itself, disposition capitalise, Whitehead definition, move with poise, philosophical foundation, cradle to grave concept, all individuals, PL not a pedagogical model. | ICSSPE Bulletin Paper |
| Whitehead | 2013 b | “This paper serves as an introduction to Len Almond’s paper on the value of physical literacy. It is one of a series of advocacy papers for use in introducing physical literacy to those less familiar with the concept.” | PL is valuable – fosters fundamental movement capacity, enhance / enrich live, develop physical potential, value of physical activity, wellbeing, give PE validity, develop motivation, confidence, self-esteem, enjoyment. | ICSSPE Bulletin Paper |
| Whitehead | 2013 c | “This short paper looks at the age related stages through which a physical literacy journey will pass and suggest some basic characteristics within each stage. Significant others who will play a part in supporting the individual in making progress are identified and the situations and contexts in which physical literacy can be nurtured indicated.” | Stages through PL journey, significant others, preschool, foundation / primary, secondary, early adulthood, older adult years, adult years, motor ability, journey is unique, everyone pass through different phases according to age, movement competence. | ICSSPE Bulletin Paper |
| Whitehead | 2013 d | “This article looks at the history of the development of the concept and makes reference both to what are seen as current attitudes to physical activity and also to the philosophy that underpins the concept.” | PE, physical activity, monism versus dualism, monism is indivisible whole, phenomenology, throughout life, sedentary lifestyles increase, existentialists – experience with interacting with the world. | ICSSPE Bulletin Paper |
| Whitehead | 2013 e | “This paper locates the issue of curriculum content within the philosophical roots of physical literacy in relation to the importance of interaction with the environment. It sets out briefly the concept of movement patterns and their development and argues for the importance of a broad and balanced experience in a range of Movement Forms. Finally the paper suggests an alternative approach to the use of extra-curricular time.” | Curriculum content, embodied dimension, movement patterns, general, refined, specific, in relation to PE curriculum, interaction with environment, building blocks of physical competence, movement capacity, to movement forms. | ICSSPE Bulletin Paper |
| Whitehead & Almond | 2013 | “This paper locates the issue of curriculum content within the philosophical roots of physical literacy in relation to the importance of interaction with the environment. It sets out briefly the concept of movement patterns and their development and argues for the importance of a broad and balanced experience in a range of Movement Forms.” | Rewarding / enjoyable experiences – enhance motivation, positive effect on confidence/ self-worth, empower to make decisions purposeful pursuits, movement capacity, experiences appreciate physical activity lifelong, practitioner good practice examples. | ICSSPE Bulletin Paper |
| Giblin, Collins & Button | 2014 | “If PL is as important as claimed then a robust empirical evidence base would seem long overdue. Accordingly, we present a critical consideration of the evaluation of PL, in order to examine options for enhancing the evidence base.” | Physical literacy, physical education, physical activity, sports participation, healthcare, physical wellbeing, psychological wellbeing, work-force productivity, limitations in movement batteries, measurement, interventions, no robust empirical tool for PL, existential and phenomenological philosophies, enriching, embodied experiences, policies offer vague guidelines, enhance evidence base, lifelong participation, Whitehead’s model, behavioural, psychological, physical, perceived competence, enjoyment, goal setting, reflection, movement capacities, assessment, self-regulated, gross motor coordination, product / process focused assessments, exergaming. | Sports Medicine |
| Roetert & Jefferies | 2014 | “The purpose of this article is to provide some background, which we hope will spark further professional discussion about the potential greater adoption of physical literacy as a key component in our field.” | Physical literacy, United States, physical education, physical activity, literacies, health literacy, computer literacy, literacy is: knowledge and understanding, thinking, communication, application, more than reading and writing, Whitehead definition, nature as human beings, sport performance, holistic, move creatively and efficiently, competence and confidence, mind and body adapt, lifespan, self-esteem, self-confidence, teachers, coaches, parents. | The Journal of Physical Education, Recreation and Dance |
| [Weiler,](javascript:__doLinkPostBack('','ss~~AR%20%22Weiler%2C%20Richard%22%7C%7Csl~~rl','');) [Allardyce,](javascript:__doLinkPostBack('','ss~~AR%20%22Allardyce%2C%20Sam%22%7C%7Csl~~rl','');) [Whyte](javascript:__doLinkPostBack('','ss~~AR%20%22Whyte%2C%20Gregory%20P.%22%7C%7Csl~~rl','');) & [Stamatakis](javascript:__doLinkPostBack('','ss~~AR%20%22Stamatakis%2C%20Emmanuel%22%7C%7Csl~~rl','');) | 2014 | “Schools are the cornerstone of nurturing fitter, happier, healthier children; however, their task would be greatly aided by cross party debate, cross party support and a government childhood physical education and physical activity policy... To continue to focus on traditional academic subjects and grades at the expense of physical literacy is short sighted, naive and demonstrates a lack of understanding of basic human behaviour and developmental needs.” | Academic performance link with physical fitness, wealthier children benefit from initiates, physical activity – priority for all schools, whole school strategies, PL equal important as reading and writing in Wales, pragmatic strategy, physical activity guidelines for children, obesity/health stats, lack of PA a form of child neglect?, government London 2012 promises, no PL definition, PE lessons not good standard, importance of schools to a healthy nation. | Journal of Sports Medicine |
| Castelli, Barcelona & Bryant | 2015 | “The primary purpose of this review is to outline the evolution of literacy among educational settings in the US.” | Educational system, physical activity, personal, environmental, behavioural, sedentary behaviour, lifespan, physical education, disposition, literacy in education, goal of PE is PL, knowledge, skills, confidence, enjoy, healthful activity, MVPA, academic benefits, health benefits, ipsative assessment, embodiment, motivation, confidence, knowledge and understanding and physical competence are interdependent constructs, on-going process, school-aged children, parental involvement, physical, cognitive, affective, culture, holistic, effective to track pupil progress. | Journal of Sport and Health Science |
| Chen | 2015 | “I will briefly discuss sources of motivation for physical activity. Then I will focus on the importance of helping children embody motivation from a perspective that children’s motivation for physical activity is both an innate mental disposition and an acquired/learned attribute. Lastly I will argue motivation cannot be embodied by itself; it must be acquired along with the development of competence in both cognitive and physical domains.” | Physical literacy, mind and body, movement, Whitehead, motivation, children, physical education, disposition, physical activity, skilfulness, efficiency, intelligence about the environment, confidence, motivation central to concept, self-efficacy, self-determination, ability to succeed, perceived competence, interest in activities, autonomy, competence, and relatedness, intrinsic/ extrinsic, physical education, competence, self-regulation. | Journal of Sport and Health Science |
| Dudley | 2015 | “The aim of this article was to present a unique conceptual model of observed physical literacy and establish an assessment rubric on which future assessment protocols may be based.” | Quality PE framework, limited knowledge and assessment, Whitehead, disposition, embodied, physical competence, knowledge and understanding, purposeful physical pursuits, lifespan, teaching games for understanding, sport education model, cognitive, affective and psychomotor, individual journey, manifestations of observed personal physical literacy, holistic, knowledge, skills, understanding, values, movement competencies, rule tactics and strategies of movement, motivational and behavioural skills, personal and social attributes of movement, physical, cognitive, affective, movement competencies. | Physical Educator |
| Ennis | 2015 | “In this paper, I will first review the theoretical basis for knowledge, transfer, and innovation as essential criteria for physical literacy.” | Literate, knowledge, skills, apply, knowledge at the heart of physical literacy, 1930 addressed PL, curricular, physical education, lifelong process, deep understanding, confident, cognitive application, content specific knowledge, enjoyable, performance, children, teacher’s role, apply knowledge into practice, knowledge based-curricular, fitness, nutrition. | Journal of Sport and Health Science |
| Hastie & Wallhead | 2015 | “The purpose of this paper was to provide a discussion of how the pedagogical features of a contemporary pedagogical model, Sport Education (SE), may be used to operationalize PL in PE and what empirical evidence currently exists to validate this claim.” | Physical literacy, embodied, physical education, disposition, physical activity, lifelong adherence, Whitehead, motivation, confidence, physical competence, knowledge and understanding, lifecourse, journey, monist, holistic, quality of life, movement with poise and economy, reading the environment, sense of self, sport education, motivation and confidence to capitalize on innate movement potential, part of a team. | Journal of Sport and Health Science |
| Jurbala | 2015 | “A main purpose of this article is to see whether there is an understanding of the concept that can encompass these disparate uses, and allow it to be theoretically  understood, practically researched, and instrumentally employed” | Physical literacy, education, sport, policy and practice, Canada, metaphoric rather than theoretic foundation, holistic mind-body connectedness, Whitehead, embodiment, sport participation, movement, community sport strategies, physical education teacher training, little empirical evidence, assessment protocols, fundamental movement skills, embodiment, quality of life, existential, phenomenological, monist, mind-body, poise and economy, reading the environment, self-esteem, self-confidence, motivation, confidence, public health, sport, interaction with the world, physical education, sport coaching pedagogy, fundamental movement skills, PL more than a metaphor. | Quest |
| Lounsberry & McKenzie | 2015 | “In our paper, we illustrate the similarity of the terms physically educated and physically literate and essentially, from a definitional perspective, find little differenced but are these terms interchangeable?” | Physically literate, physically educated, team building, curriculum integration, movement education, sport education, USA, motivation, confidence, physical competence, knowledge and understanding, more than reading and writing, physically educated variety of activities, physically fit, values participation, physically literate and educated non synonymous as used in K-12 national standards, Whitehead. | Journal of Sport and Health Science |
| Lundvall | 2015 | “The purpose of this paper is, through an explorative  literature overview, to explore frequent and significant themes of physical literacy” | Literacy, reading and writing, communication, understanding, beyond reading and writing, social practices, physical education, physical literacy, Whitehead, physical activity, educative role, philosophical ideas. children and youth, PL to develop PE practice, fundamental movements as building blocks, self-referenced, motivation, inclusive, pedagogy, sports development, PL not synonymous to FMS, social influences, assessment, summative tension, mastery, knowledge, embodied motivation, assessment batteries, CAPL, scepticism about assessing PL, goal-oriented assessment driven school systems, competence rather than performance code, driven by pedagogy. | Journal of Sport and Health Science |
| MacDonald | 2015 | “The purpose of this article is to help high school physical education teachers move students toward physical literacy by illustrating how the new grade-level outcomes can be implemented.” | Physical education, high school, teachers, student engagement, passively engaged in PE, perceived competence, choice of activity, intrinsic motivation, confidence, motor skills competency, mastery orientated tasks, reduce social comparisons, knowledge and skills, throughout adulthood, fitness and physical activity plans, no PL definition/ explanation. | The Journal of Physical Education, Recreation and Dance |
| Roetert & MacDonald | 2015 | “The purpose of this paper is to “unpack” the concept of physical literacy, its role in PE, and the potential ramifications for K-12 teachers and learners.” | Physical literacy, integrated into policies, global, health, physical education, physical activity and inactivity, obesity, ramifications for K-12 teachers and learners, Whitehead, philosophical foundation, monist, whole, not dualistic, US, move with confidence and competence, variety of activities, development of the whole person, relationship between PL and PE, outcome of PE is PL, embodiment, teachers, equal to other areas of the curriculum, positive attitude, sense of achievement, enjoyment, movement competence, self-awareness, movement patterns, operationalizing PL in PE, lifetime activity, skill competency, motor learning, engage students, modified and small sided tasks, differentiation instruction, systematic assessment to track individual progress. | Journal of Sport and Health Science |
| Silverman & Mercier | 2015 | “The purpose of this paper is to provide an overview of instructional design decisions that teachers make where research suggests that motor skill learning can be enhanced.” | Physical education, teachers, motor skills, competent, confident, motivation, knowledge, attitudes, lifelong participation, psychomotor, cognitive, affective, monism, instructional designs, fundamental movement skills, physical activity, time spent correlated with motor skill achievement, task organization. | Journal of Sport and Health Science |
| Sprake & Walker | 2015 | “This paper concludes that a number of aspects require  further consideration if the true value of Physical Education is to be realised.” | National curriculum, PE, PE develop child holistically, support academic achievement, dualistic, holistic, marginalised PE, perceived importance, elitism, social inclusion, health and economic regeneration, motivation, confidence, physical competence, knowledge and understanding, Whitehead, PE academic subject, lifespan, physical activity, disposition, monist approach, existentialism, phenomenology, academic achievement, | European Physical Education Review |
| Sun | 2015 | “This article reviews findings from research and critique research on AVGs in light with the theoretical and pedagogical tenets of physical literacy and, on the basis of the review, elaborates the potential that AVGs could contribute to enhancing children’s physical literacy.” | Mind-body, holistic approach to physical activity, cognitively knowledgeable, physically competent, motivated, throughout the lifespan, active video games, confidence, value physical activity, participation, physically fit, technology in curriculum development, instructional design and assessment of PE, embodied, exergames, body movement, motivated, situational interest, enjoyment, social interaction, motivation may drop over time with AVG’s, Whitehead. | Journal of Sport and Health Science |
| Corbin | 2016 | “This article provides a unique overview on the topic and asks specific questions about the benefits and problems associated with the use of the term physical literacy.” | Physical education, developing whole child, literacy, educated, cultured, ability to read and write, sport-based organization, philosophy, purpose, expertise, motor skills, cognitive skills, physical activity, physical fitness, values physical activity, interaction with others, motivation, confidence, perception of environment, responsibility, engagement for life, assessment of physical literacy easy to administer. | Research Quarterly for Exercise and Sport |
